# In vitro conversion of ellagic acid to urolithin A by different gut microbiota of urolithin metabotype A

**DOI:** 10.1007/s00253-024-13061-1

**Published:** 2024-02-16

**Authors:** Fuxiang He, Yingying Bian, Yaling Zhao, Mengjie Xia, Shu Liu, Jiajin Gui, Xiaoyue Hou, Yaowei Fang

**Affiliations:** 1 Jiangsu Key Laboratory of Marine Bioresources and Environment /Jiangsu Key Laboratory of Marine Biotechnology, Jiangsu Ocean University, Lianyungang, 222005 China; 2 Co-Innovation Center of Jiangsu Marine Bio-Industry Technology, Jiangsu Ocean, Lianyungang, China; 3 College of Ocean Food and Biological Engineering, Lianyungang, 222005 China

**Keywords:** Gut microbiota, Ellagic acid, Urolithin A, In vitro fermentation, High throughput sequencing

## Abstract

**Abstract:**

The metabolite urolithin A, a metabolite of the dietary polyphenol ellagic acid (EA), has significant health benefits for humans. However, studies on the gut microbiota involved in ellagic acid metabolism are limited. In this study, we conducted in vitro fermentation of EA using human intestinal microbiome combined with antibiotics (vancomycin, polymyxin B sulfate, and amphotericin B). Liquid chromatography-mass spectrometry (LC–MS/MS) analysis demonstrated that the production capacity of urolithin A by gut microbiota co-treated with polymyxin B sulfate and amphotericin B (22.39 µM) was similar to that of untreated gut microbiota (24.26 µM). Macrogenomics (high-throughput sequencing) was used to analyze the composition and structure of the gut microbiota. The results showed that the abundance of *Bifidobacterium longum*, *Bifidobacterium adolescentis*, and *Bifidobacterium bifidum* in the gut microbiota without antibiotic treatment or co-treated with polymyxin B sulfate and amphotericin B during EA fermentation was higher than that in other antibiotic treatment gut microbiota. Therefore, *B. longum*,* B. adolescentis*, and *B. bifidum* may be new genera involved in the conversion of EA to urolithin A. In conclusion, the study revealed unique interactions between polyphenols and gut microbiota, deepening our understanding of the relationship between phenolic compounds like EA and the gut microbiota. These findings may contribute to the development of gut bacteria as potential probiotics for further development.

**Key points:**

• *Intestinal microbiome involved in ellagic acid metabolism.*

• *Gram-positive bacteria in the intestinal microbiome are crucial for ellagic acid metabolism.*

• *Bifidobacterium longum, Bifidobacterium adolescentis, and Bifidobacterium bifidum participate in ellagic acid metabolism.*

**Supplementary Information:**

The online version contains supplementary material available at 10.1007/s00253-024-13061-1.

## Introduction 

A complex microbial community having various physiological functions exists in the human gut. The gut microbiome consists mainly of strictly anaerobic bacteria (Grech et al. 2021). Phylogenetically, approximately 90% of intestinal bacteria belong to *Firmicutes* and *Bacteroidetes*, while the remaining microbiome belong to *Actinobacteria*, *Proteobacteria*, *Fusobacteria*, and *Verrucomicrobia* (Singh and Natraj 2021). The composition and structure of the gut microbiota are unique to every individual and vary throughout an individual’s life. The intestinal microbiota is a crucial player in influencing host immunity, digesting food, regulating intestinal endocrine function, modifying drug action, and removing toxins (Zhao et al. 2021). Gut microbes perform various intestinal functions, including shaping the host’s intestinal mucosa, improving immune system maturation, and contributing significantly to the nutrient absorption level (Pineiro-Ramos et al. 2021; Ray and Mukherjee 2021). Intestinal microbes have also established critical links with various physiological and pathological conditions, including extraintestinal diseases, cardiac metabolism, and cardiovascular diseases (Wu et al. 2015). Feces are believed to reflect this intestinal microbial diversity and can be regarded as a reservoir of microbes associated with the human gastrointestinal tract (GIT) (Gaci et al. 2017). The sources of metabolic substrates for the human gut microbiota are exogenous and endogenous, and the metabolic functions of this microbiota depend on the nature of colonic substrates available for fermentation (Duarte et al. 2021). Therefore, understanding how the gut microbiome contributes to human health and nutrition is crucial for developing preventive nutrition strategies for various health conditions.

Ellagic acid (EA) is a natural polyphenol widely present in edible and medicinal plants such as fruits, nuts, and tea. It is mainly present in its condensed form, that is, ellagitannins (ETs) (Djedjibegovic et al. 2020). ETs have various health effects, but their absorption by the GIT is difficult (Zhang et al. 2022a). However, ETs and EA are metabolized by gut microorganisms to form urolithins, which are readily absorbed by GIT. Urolithins have some biological activities. Among all urolithins, urolithin A (Uro-A) exhibited the most biological activity, an excellent safety, and can be detected in the micromolar concentration range in the plasma (Al-Harbi et al. 2021; Heilman et al. 2017). Moreover, Uro-A stimulates mitochondrial autophagy, thereby improving mitochondrial and skeletal muscle health. Depending on the urolithin types formed, three types of urolithin metabolism (UM) have been described, UM-A, UM-B, and UM-0 (Romo-Vaquero et al. 2019). The end product of UM-A metabolism is Uro-A production, and UM-B metabolism produces Uro-B in addition to Uro-A.

As a result, determining the gut microbiome involved in EA conversion and exploring the relationship between EA metabolism and gut microbiome have become a focus of research worldwide. EA alters the intestinal microbiome by increasing beneficial bacteria (e.g., *Lactobacillus* and *Bifidobacterium*) and decreasing harmful bacteria (e.g., *Clostridium* and *Escherichia coli*) as well as other bacteria involved in the intestinal microbiome (*Bacillus* spp., *Eubacterium* spp., and *Enterococcus* spp.) (Mithul Aravind et al. 2021; Ozdal et al. 2016). At present, numerous studies have reported the important influence of intestinal microbiome on EA transformation, and several bacterial strains that can cause this transformation have been isolated separately. Two strains of urolithin-producing bacteria, *Gordonibacter urolithinfaciens* DSM27213^T^ and *Gordonibacter pamelaeae* DSM19378^T^, were identified by Spanish authors (Selma et al. 2014a, b). A study for the first time confirmed the urolithin-producing capacity of a pure strain. In that study, *Ellagibacter isourolitinifaciens* DSM104140^T^ was a IsoUro-A-producing strain, and *Bifidobacterium pseudocatenulatum* INIA P815 was the other Uro-A-producing strain (Gaya et al. 2018). In addition, Uro-A-producing strains were isolated from human feces in our laboratory (e.g., *Enterococcus faecium* FUA027 (Xia et al. 2023; Zhang et al. 2022c) and *Lactococcus garvieae* FUA009) (Mi et al. 2022).

Macrogenomic 16S rRNA sequencing also revealed that *Akkermansia* was more prevalent in fecal samples from individuals who could convert pomegranate extracts to Uro-A (Henning et al. 2017). On feeding polyphenol-rich peach and plum extracts to obese rats, the abundance of *Bacteroidetes*, *Enterococcus faecalis*, and *Lactobacillus* significantly increased in the GIT of these rats (Luo et al. 2023). Although some progress has been made in the study of urolithin-related strains, studies exploring the specific groups of intestinal bacteria that are involved in EA transformation are few.

In this study, Gram-positive and Gram-negative bacteria and fungi from the intestinal microbiome of UM-A volunteers (individuals whose gut microbiota is able to metabolize EA to Uro-A are defined as UM-A volunteers) were inhibited in vitro experiments by vancomycin, polymyxin B sulfate, and amphotericin B, respectively (Kohanski et al. 2010; Nunziata et al. 2022), and different intestinal microbiomes were obtained for the EA-converting in vitro fermentation assay. Liquid chromatography-mass spectrometry (LC–MS/MS) was used to analyze the transformation products of EA metabolized in vitro by the intestinal bacterial population of UM-A volunteers. High-throughput sequencing techniques, 16S rRNA were applied to analyze the effects of EA on the composition of the human intestinal microbiome. These findings helped us to identify the types of intestinal microbiome that could potentially transform EA and to explore the correlation between changes in microbial populations and final metabolites during EA fermentation.

## Materials and methods

### Chemicals

EA powder (purity ≥ 99%), urolithin C (purity ≥ 98%), Uro-A (purity ≥ 99%), and amphotericin B were purchased from Sigma (Sigma-Aldrich, St. Louis, MO, USA). Vancomycin, polymyxin B sulfate, ethyl acetate, and liquid paraffin were purchased from Macklin Biochemical (Shanghai, China). Formic acid and acetonitrile were purchased from Aladdin Chemical Company (Shanghai, China), and brain heart leachate broth (BHI) was purchased from OXOID (Basingstoke, UK). All other chemicals used are of analytical and chromatographic grade.

### Collection of fecal samples from volunteers

The volunteers (22–27 years old) should not have taken any antibiotic-related medications for at least 3 months and should have no history of gastrointestinal diseases. All volunteers signed a written informed consent. On the day of the experiment, sterile tubes were used to collect fresh stool samples from these volunteers. The collected fecal samples were transferred to a centrifuge tube containing 20 mL of 0.9% sterile saline and homogenized. Then, they were centrifuged at 200 × *g*. The resulting supernatant was used as an inoculum for fecal microbiome.

### Culture conditions and pre-culture treatment

Brain heart leachate broth (BHI) used as a culture medium under anaerobic conditions was made by sealing with paraffin oil according to Gaya et al. (2018). The substrate EA (15 mM) was dissolved in propylene glycol in advance, added at 1:1000 (v:v) to a 15-mL autoclave-resistant glass anaerobic tube containing 10 mL of BHI medium, and sterilized at 121 °C for 20 min in an autoclave. The medium was equilibrated in an anaerobic tank AG0025A (OXOID, Basingstoke, UK) at room temperature for at least 12 h before the fecal suspension was completely prepared.

### Antibiotic-treated intestinal microbiome and EA conversion experiments in vitro

Antibiotics were used at the following concentrations to treat the intestinal microbiome: amphotericin B, 10 mg/L; vancomycin, 10 mg/L; and polymyxin B sulfate, 62,000 IU/L (Stevenson et al. 2000).

Three antibiotics are combined in pairs to treat the intestinal microbiota. The antibiotic-treated gut microbiota served as the experimental group, while the intestinal microbiome was untreated as the control. The experimental group P_A represents the combined treatment of polymyxin B sulfate and amphotericin B. The experimental group P_V represents the combined treatment of polymyxin B sulfate and vancomycin. The experimental group A_V represents the combined treatment of amphotericin B and vancomycin. The control group was inoculated with 1% suspension of UM-A enteric microbiome in 10 mL BHI (pH 7.4) containing 0.5 g/L L-cysteine (Ourchem, Guangzhou, China) and 15 µM EA. Unlike the control group, antibiotics were added to the culture solution in the experimental group. In addition, we set a blank group corresponding to each experimental group. Compared with the experimental group, the culture medium of the control group does not contain EA. All cultures were incubated for 1–3 days under anaerobic conditions at the Whitley DG250 Anaerobic Workstation (Don Whitley Scientific, Shipley, UK) at 37 °C. Three independent replicate experiments were conducted for each group.

### Extraction of metabolites

Two milliliters of fermentation broth from 0, 24, 48, and 72 h of in vitro fermentation was extracted using an equal volume of ethyl acetate containing 1.5% formic acid. The broth obtained was inactivated at 121 °C and vortexed. The extracted samples were evaporated to dryness at room temperature under a stream of nitrogen. The precipitates were re-dissolved with 600 µL of organic solvent (C_2_H_3_N: H_2_O: H-COOH = 80: 19.9: 0.1) and volume fixed. The supernatants were then filtered through 0.25-μm polyvinylidene fluoride (PVDF) filter membranes into a brown injection bottle for later use.

### High-throughput sequencing of 16S rRNA

16S rDNA amplicon sequencing is a vital tool for studying the microbial community composition in environmental samples. The aim is to select several regions of variation, design universal primers for PCR amplification of conserved regions, and sequence the highly variable regions for analysis and strain identification (Shi et al. 2022).

#### Sequencing procedure

To characterize the bacterial community structure by performing 16S rRNA gene sequencing, the samples were lyophilized and the genomic DNA was extracted using the sodium dodecyl sulfate (SDS) method. The extracted DNA was stored in DNA centrifuge tubes. The samples were diluted to 1 ng/µL with sterile water and used as a template for PCR, which was performed using 16S V4 primers (515F and 806R) (Apprill et al. 2015; Parada et al. 2015). Then, the construction of small fragment libraries was followed by double-end sequencing. Total DNA extraction, PCR amplification, and Illumina HiSeq sequencing were performed with the assistance of Wuhan Mavis Metabolic Biology (www.metware.cn).

#### Data processing

The sample data were data spliced and filtered to obtain usable Tags data (Clean Tags). Quality control and noise reduction operations were performed on the Tags by referring to the QIIME analysis of sequencing results (v1.9.1, http://qiime.org/scripts/split_libraries_fastq.html). The raw sequencing data are available on NCBI SRA PRJNA1013743.

### Analysis of intestinal microbiome diversity and comparison of abundance

The valid data of each sample were first clustered using operational taxonomic units (OTUs) based on the 97% sequence similarity principle. Then, the OTU sequences were annotated with species to determine the species composition of each sample. The diversity of the gut microbial community (including *α*-diversity and *β*-diversity) was detected at each taxonomic level (phylum, class, order, family, genus, species) by using R software (Lucent Technologies, Murray Hill, NJ, USA). Based on relative abundance, the Shannon index was calculated for each sample. To assess *β*-diversity among samples, the Bray–Curtis dissimilarity matrix was measured and ranked using multidimensional scaling. Species/genus relative abundance of > 1% was selected to compare the abundance of the fermentation broth from the blank group (no EA), experimental group (with antibiotics and EA), and control group (with EA).

### Metabolite identification and concentration analysis

Liquid chromatography-mass spectrometry (LC–MS/MS) and high-performance liquid chromatography (HPLC) were used to characterize and quantify the product, respectively.

#### High-performance liquid chromatography determination

The metabolites were quantified qualitatively using an Agilent 1290 Infinity II LC system (Technologies. Santa Clara, CA, USA). The chromatographic conditions were as follows: liquid chromatographic column TC-C_18_ (4.6 × 250 mm, 5 µm 588925–902, Waters, Wilmslow, UK), column temperature of 30 ℃, mobile phases A and B were milli-Q water (0.1% formic acid) and acetonitrile (0.1% formic acid), respectively, flow rate of 0.3 mL/min, and an injection volume of 10 µL. The gradient elution used was as follows: 0–15 min, 20–70% mobile phase A; 15–20 min, 70–95% mobile phase A; 20–21 min, 95–100% mobile phase A; 22–24 min, 100–20% mobile phase A; 24–25 min, 20% mobile phase A. Data acquisition and processing were performed using Agilent Chemistry Workstation (Technologies. Santa Clara, CA, USA). All metabolites were quantified at 305 nm by using their standards at 15 µM for comparison.

#### Urolithin of liquid chromatography-mass spectrometry (LC–MS/MS) analysis

An Agilent 1290 Infinity II liquid chromatography system and an Agilent 6550 QTOF mass spectrometer (Waters, Milford, MA, USA) were used for sample analysis. The extracts were eluted using water containing 0.1% formic acid (A) and acetonitrile (B) with 0.1% formic acid on a high-performance liquid chromatography (HPLC) column containing 1.7-μm particles (2.1 × 100 mm ACQUITY UPLC BEH C_18_ column) at 0.35 mL/min a flow rate. The gradient (expressed as A%) procedure used was as follows: 0–0.5 min, 95% A; 0.5–20 min, 95–5% A; and 2 min, 5% A. The column temperature and injection volume were 40 °C and 2 µL, respectively. Mass spectrometry analysis was performed using the negative ion mode of the electrospray source and mass spectrometry acquisition mode with a selected mass range of 50–1200 m/z. The ionization parameters used were as follows: capillary voltage, 2.5 kV; cone voltage, 30 V; source temperature, 120 °C; desorption gas temperature, 400 °C; and collision energy, 20–40 eV.

### Correlation analysis

To determine the relationship between the overall microbiome and Uro-A, metabolite peak areas were mapped as factors to the ranking of abundance distribution maps of intestinal microbiome after the microbiome was treated with antibiotics. Correlations between affected microbial species and urolithins were determined by conducting the Spearman correlation test by using the R program package (University of Auckland, New Zealand).

### Statistical analysis

For the optimization of extraction parameters, GraphPad Prism (San Diego, CA, USA) was used to determine significance by analyzing *F*-values at *P* < 0.05 for statistical analysis. The *α*-diversity index between-group variance analysis was performed using *t*-test, Tukey, and Kruskal–Wallis tests to analyze whether species diversity between the groups was significantly different. The *β*-diversity group differences were analyzed using *t*-test, Tukey, and Kruskal–Wallis tests to determine significant differences in species diversity between the groups. All samples were measured three times in parallel, with data being expressed as the mean ± standard deviation (SD).

## Results

### Analysis of EA in vitro fermentation products

In this study, seven volunteers provided their fecal samples, three of whom (No.4–6) had gut microbiome capable of metabolizing EA to produce Uro-A, indicating that these three individuals were UM-A populations (Supplemental Fig. S1). Compared to the other 2 UM-A volunteers, the sixth volunteer had approximately twice the level of urolithin A-producing. The fecal sample used in subsequent research was collected from the sixth volunteer (22 years old) who was one of the three UM-A volunteers.

**Fig. 1:**
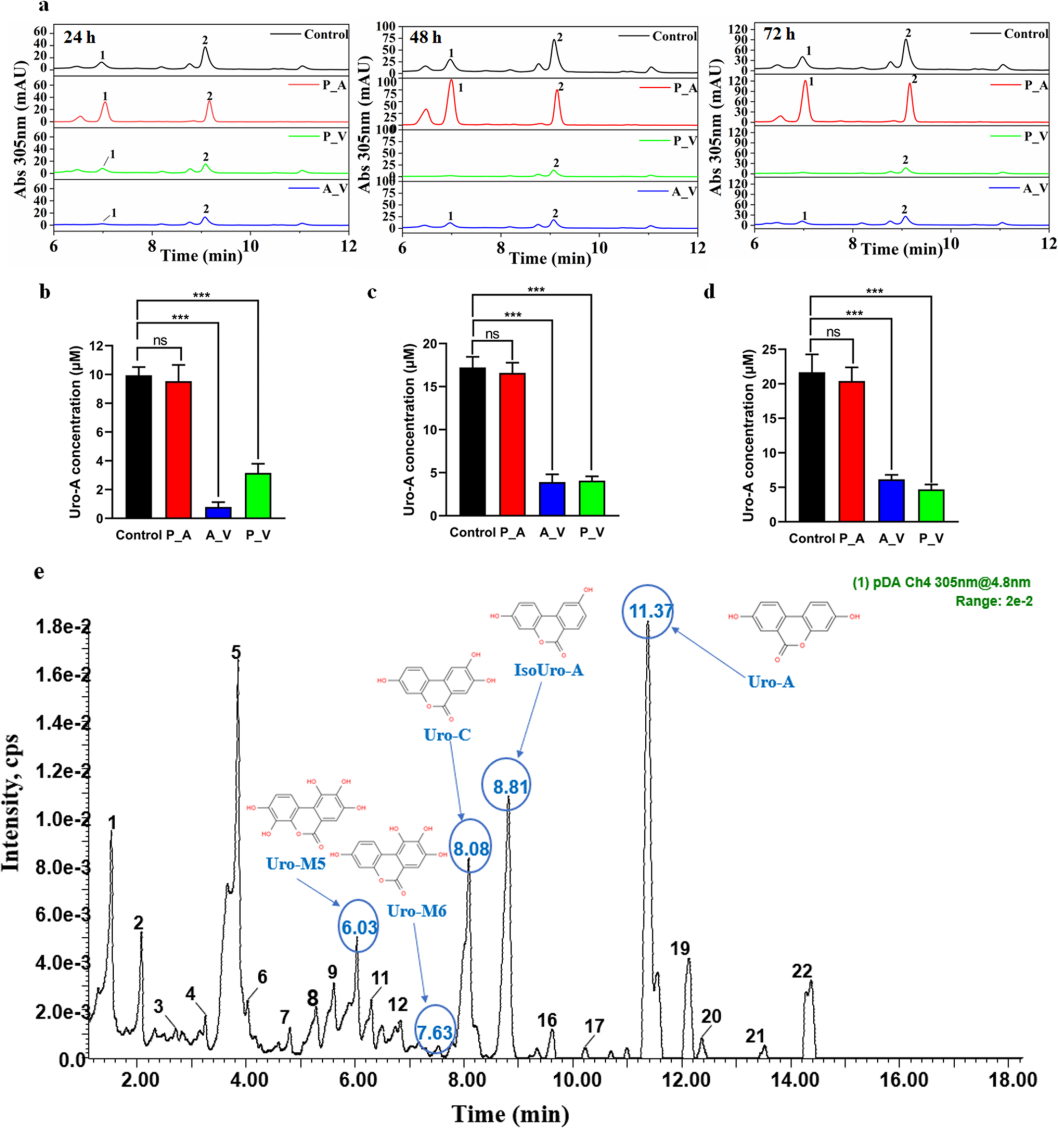
Effect of antibiotics treatment on the conversion of EA to generate Uro-A by intestinal microbiome in a UM-A volunteer. **a** HPLC analysis of EA in vitro fermentation products. 1 and 2 represent Uro-C and Uro-A, respectively. **b**–**d** Uro-A concentration in the fermentation broth of P_A group fermented for **b** 24 h, **c** 48 h, and **d** 72 h. Significant differences from the controls are shown as *P < 0.05, **P < 0.01, and ***P < 0.001. ns, no significant. **e** Summary plot of HPLC–MS/MS of the fermentation broth of P_A group fermented for 48 h. cps, stands for counts per second, which is the number of ions produced per second by the mass spectrometer

To explore the primary group of gut microorganisms involved in the metabolism of EA for Uro-A production, we utilized a combination of two antibiotics to treat the gut microbiome. There were three antibiotic-treated groups (experimental groups) in this study. As shown in Fig. 1a–d, the results of HPLC analysis showed that Uro-A could be detected in the fermentation broth of all three experimental groups (P_A, P_V, and A_V) as well as the control group (no antibiotic treatment). Furthermore, there was no significant difference in Uro-A content between the P_A group (22.39 µM) and the control group (24.26 µM). In addition, LC–MS/MS analysis identified 22 compounds in the fermentation broth of P_A group fermented for 48 h (Fig. 1e). Five urolithins (Uro-A, Uro-C, IsoUro-A, Uro-M5, and Uro-M6.) could be identified in the fermentation broth by comparing the retention time, exact MS of m/z, and MS/MS spectra with standards (Fig. 1e and Supplemental Table S1). These results suggest that combined treatments of antibiotics polymyxin B sulfate and amphotericin B have little effect on the Uro-A production, but that combination treatments of polymyxin B sulfate and vancomycin (or amphotericin B and vancomycin) severely impair the production of Uro-A.

### Response characteristics of intestinal microbiome to antibiotics and EA

**Fig. 2:**
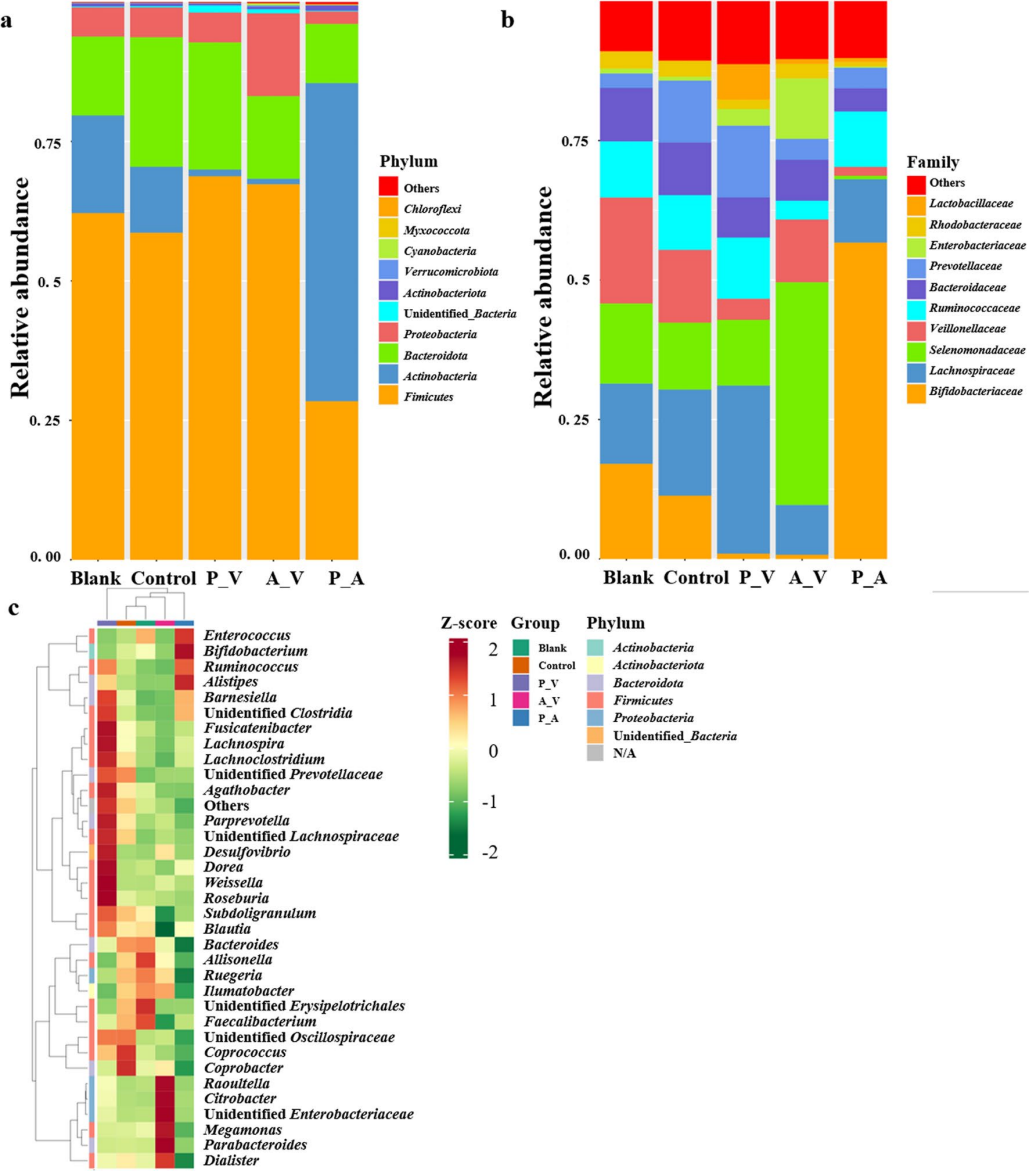
Intestinal microbiome richness in all the samples. **a**–**b** Metagenomic bacterial taxonomic distribution based on relative abundance of clean metagenomic reads at **a** phylum and **b** family level. **c** Heat map of 35 most abundant bacterial genera. N/A, not applicable

We used 16S rRNA high-throughput sequencing techniques to analyze the microbiota composition in the fermentation broth. In total, 215,1916 sequences were generated from all the samples. Following denoising and assembly, 190,2824 qualified sequences of an average of 214 bp were obtained. Based on the sum of the quantitative values of microbiota in all samples, the top 10 microbial species with maximum abundance were further selected for the principal component analysis. As shown in Fig. 2a, *Bacteroidota* (23.21%), *Firmicutes* (68.75%), *Proteobacteria* (14.86%), and *Actinobacteria* (57.03%) were dominant at the phylum level in samples from the control, P_V, A_V, and P_A groups, respectively. In addition, *Veillonellaceae* (13.04%), *Lachnospiraceae* (30.13%), *Selenomonadaceae* (39.93%), and *Bifidobacteriaceae* (56.71%) were dominant at the family level in samples from the control, P_V, A_V, and P_A groups, respectively (Fig. 2b and Supplemental Table S2). Furthermore, we here classified the 35 most abundant bacterial genera from all the samples. As shown in Fig. 2c, in the control samples, the dominant species were *Coprobacter* (1.47) and *Coprococcus* (1.42) at the genus level. However, the dominant microbiome in the P_V samples were *Dorea* (1.72), *Weissella* (1.77), and *Roseburia* (1.76)*. Raoultella* (1.73), *Citrobacter* (1.74), *Megamonas* (1.66), and *Parabacteroides* (1.76) were more heavily weighted in the A_V samples. In the P_A samples, *Enterococcus* (1.41), *Bifidobacterium* (1.70), and *Alistipes* (1.54) were dominant (Fig. 2c and Supplemental Table S3). These results suggest that the addition of antibiotics had an effect on the richness and diversity of the intestinal microbiota.

To investigate the microbiome response to the EA intervention, we compared the composition of the gut microbiome in the fermentation broth of the blank group and the control group. In the blank samples, *Firmicutes* (62.15%) were the predominant bacteria at the phylum level, followed by *Actinobacteria* (17.51%), *Bacteroidota* (14.13%), and *Proteobacteria* (5.23%) (Fig. 2a). At the family level, *Veillonellaceae* (18.99%) were the most common bacteria, followed by *Bifidobacteriaceae* (17.05%), *Lachnospiraceae* (14.38%), and *Selenomonadaceae* (14.36%) (Fig. 2b and Supplemental Table S2). However, the abundance of *Bacteroidota* increased from 14.13 to 23.21% in the control group compared to the blank group. Furthermore, the control group had a higher abundance of *Lachnospiraceae* and *Prevotellaceae* at the family level. The abundance of the two increased from 14.38% and 2.60% to 19.02% and 11.10%, respectively (Fig. 2b). These results directly reflect that *Lachnospiraceae* and *Prevotellaceae* may be involved in the metabolism of EA.

**Fig. 3:**
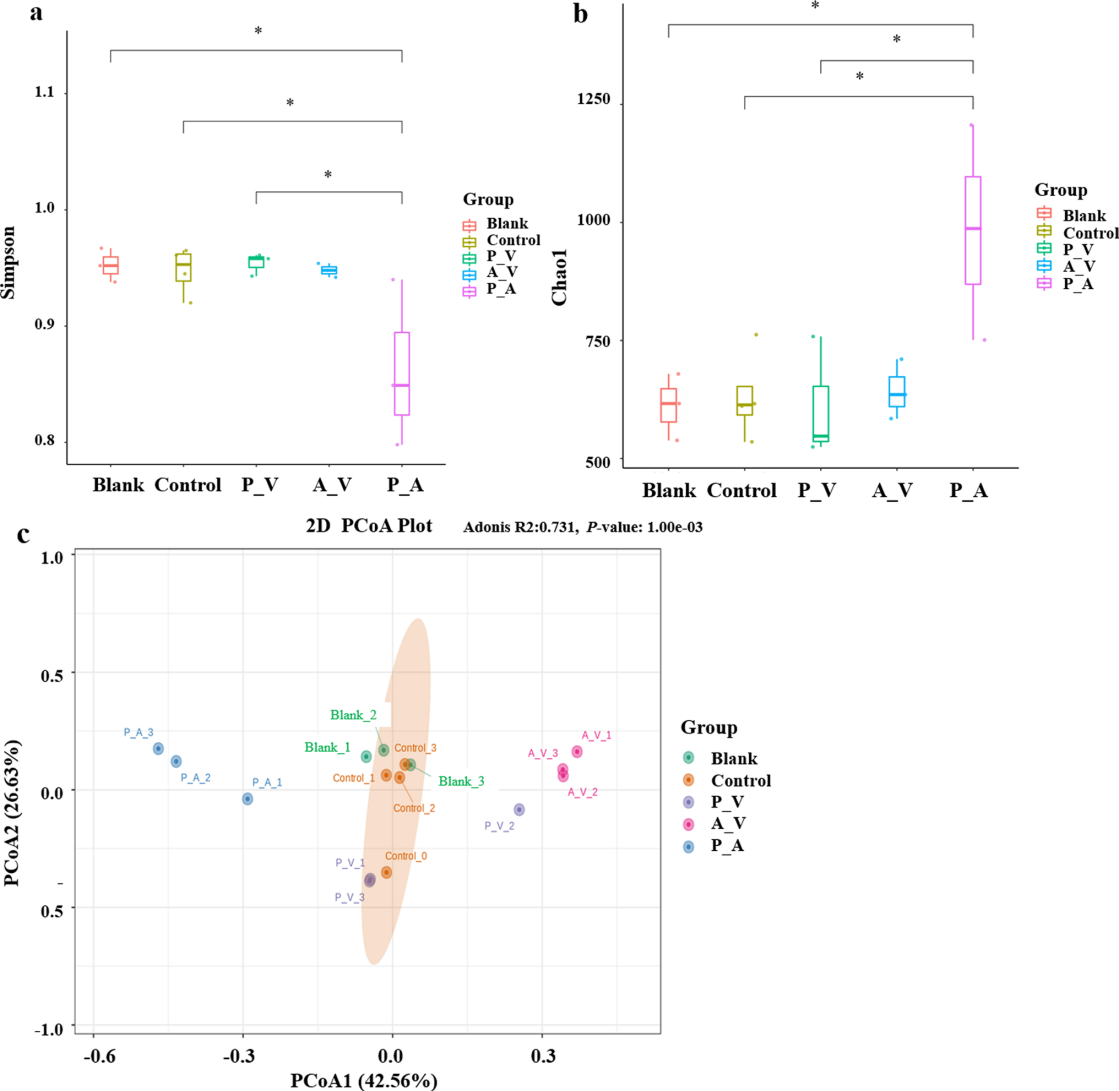
Analysis of fecal microbial diversity after EA fermentation. Bacterial *α*-diversity Simpson (a) and Chao1 indices (b) and weighted UniFrac distance PCoA (c). 1.2.3 represents 24 h, 48 h and 72 h respectively. Data are shown as the mean ± SEM, and results are considered statistically significant at *P* < 0.05. *P < 0.05, **P < 0.01, ***P < 0.001

To further analyze the effects of antibiotics on the intestinal microbiome, we analyzed the microbiome diversity of all samples. As shown in Fig. 3a and b, the species diversity of the control group was higher, but the intergroup differences of species were larger. The species diversity of the P_A group was lower, but the distribution of each species was more even. The above results indicate that antibiotics (polymyxin B sulfate and amphotericin B) directly affect the diversity of intestinal microbiota in the P_A group. Moreover, the ASV-based weighted UniFrac distance principal coordinate analysis (PCoA) was used to reveal the microbiota diversity (Fig. 3c). Compared to the control group, the overall structure of the 24 h intestinal microbiome of the A_V and P_A samples differed from that of the 48 and 72 h intestinal microbiome of these samples. This shows a displaced aggregation of bacterial composition, indicating that different antibiotics significantly alter the structure of gut microbiota.

To further elucidate inter- and intra-group differences, the similarity analysis (ANOSIM) was performed. The results (Supplemental Fig. S2a-c) revealed that *R* > 0 between the control and three experimental group samples, which indicated that the differences in *β*-diversity between the control and three experimental group samples were significantly greater than the intra-group differences. Similarly, the difference in *β*-diversity was significantly greater in the P_A group samples compared to the P_V and A_V group than in the intra-group differences (Supplemental Fig. S2d-f). Furthermore, the species clustering analysis revealed that intestinal microbial bacteria were clustered because of antibiotics (Supplemental Fig. S2g and h).

**Fig. 4:**
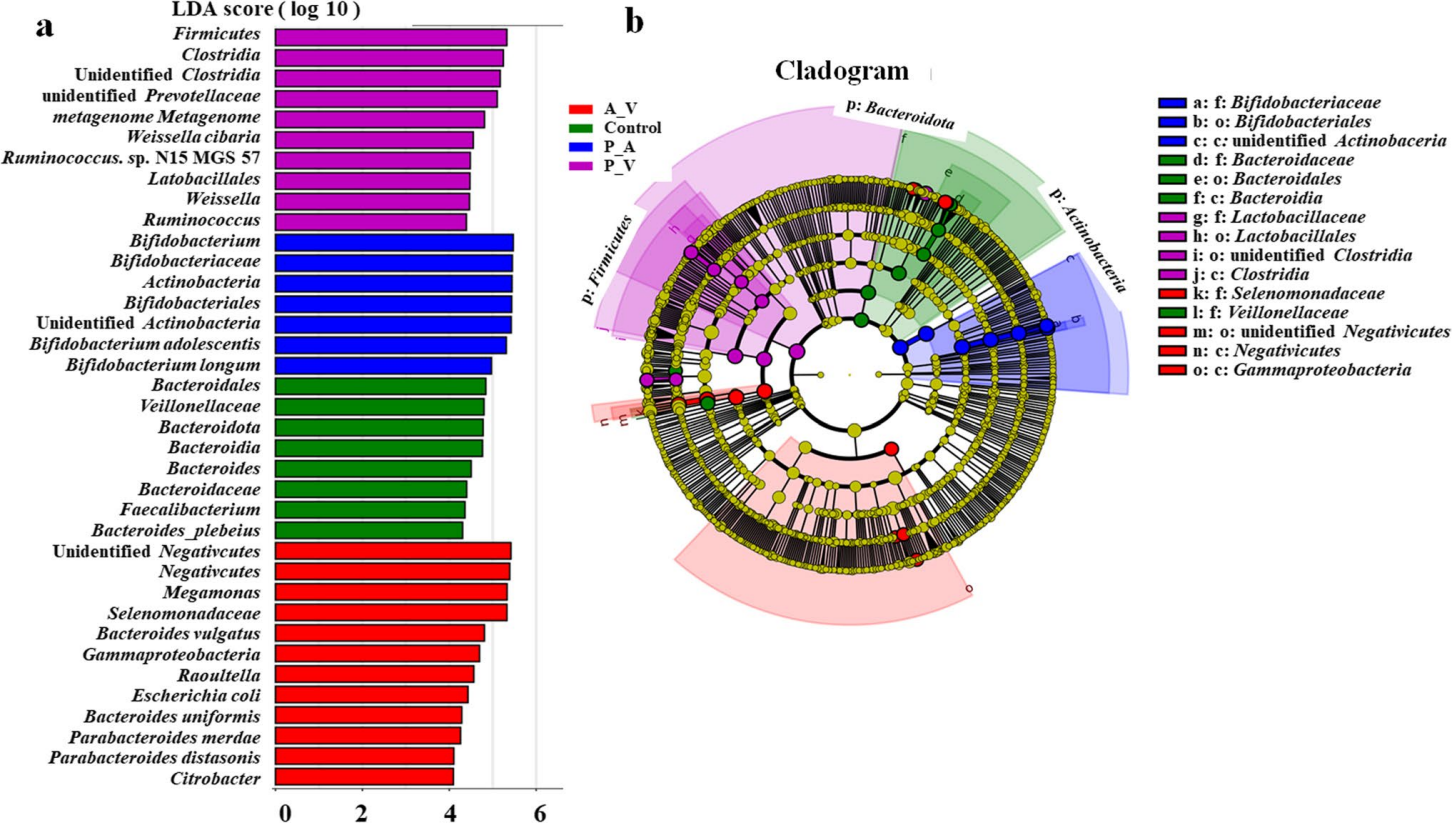
Statistical results of LEfSe. The generated LDA score (**a**) and branching plots (**b**) display the difference in the abundance of bacterial communities between the control and experimental groups. Microbial taxa exhibit LDA scores of greater than 4. Red, green, blue, and purple nodes represent taxonomic units significantly over-represented in the A_V, control, P_A, and P_V groups. The diameter of each circle is proportional to the abundance of taxonomic units

Moreover, the linear discriminant analysis effect sizes (LEfSe) were analyzed for the samples fermented for 24, 48, and 72 h. Compared with the control group, the genus *Bifidobacterium* and the species *Bifidobacterium adolescentis* and *Bifidobacterium longum* were more predominant in the P_A group at the genus level (Fig. 4a)*.* However, the abundance of *Veillonellaceae* and *Bacteroidaceae* was lower in the P_A group at the family level. The genus *Faecalibacterium* and the species *Bifidobacterium plebeius* were lower in the P_A group at the genus level. *Bifidobacteriaceae* were significantly increased in the P_A group compared to the control group (*P* < 0.05) (Fig. 4b). Notably, the initial control group samples had a low abundance of *Bifidobacterium*. The results indicated antibiotic-related differences in the relative abundance of different gut microbiota.

### Differential analysis of gut microbiota involved in EA metabolism

**Fig. 5:**
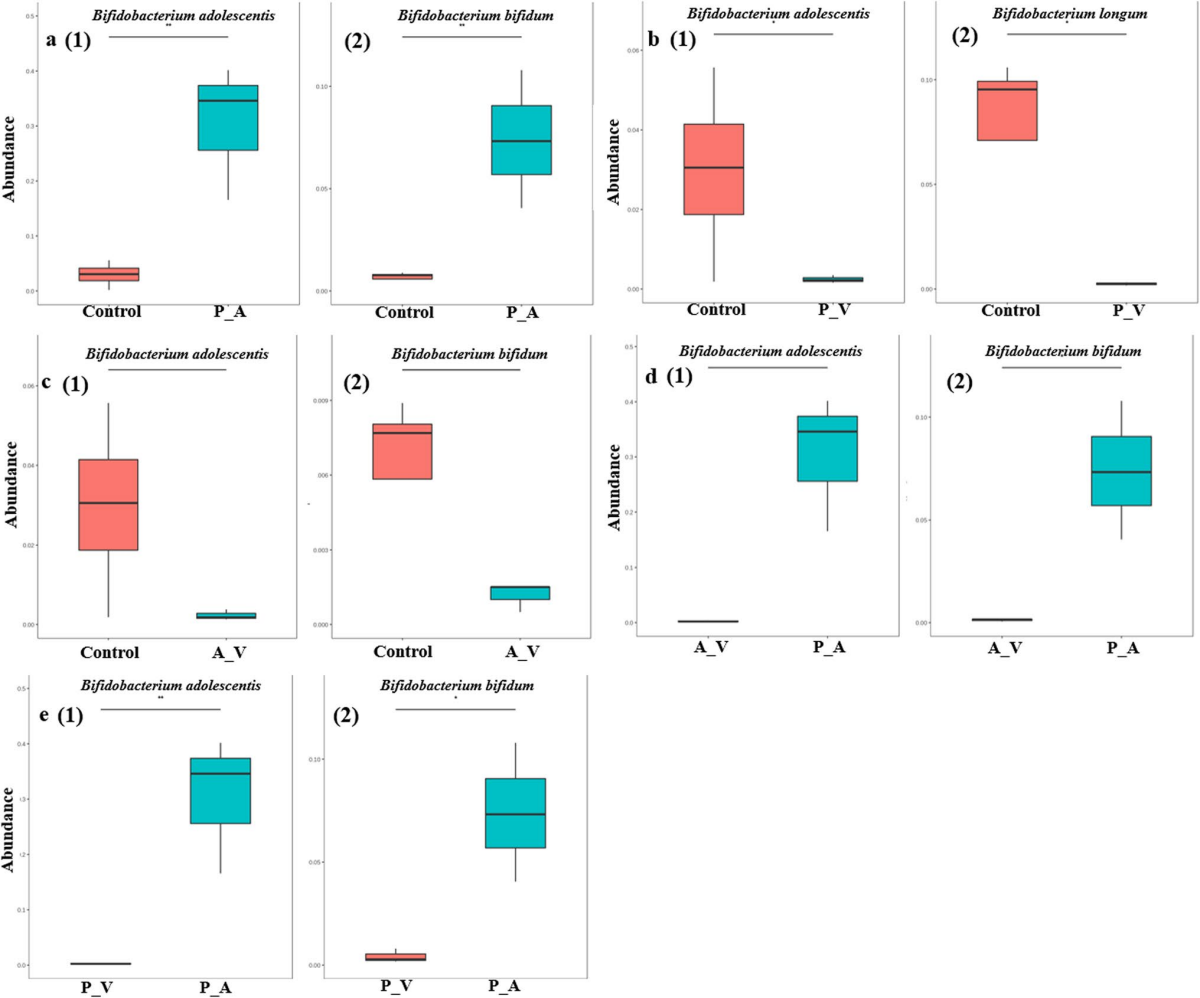
Box plot of abundance distribution of differential species between groups. **a**–**e** Bacterial differences. The horizontal axis is the sample subgroup, and the vertical axis is the relative abundance of the corresponding species. The horizontal line represents the two subgroups with significant differences. *P < 0.05, **P < 0.01

To explore the microbiome involved in EA metabolism, the species abundance distribution between groups was hypothesis tested by using the Metastats method (Youssef et al. 2009). Box plots of the abundance distribution of differential species between the groups were generated using the obtained results with significant *P* values (Fig. 5).

The abundance of *B. adolescentis* and *Bifidobacterium bifidum* in P_A group samples was significantly higher than that of the control group (*P* < 0.05), but there were no significant differences in Uro-A levels between these two groups (Fig. 5a). The differential microbiota between the P_V samples and the control samples included *B. adolescentis* and *B. longum*, and the differential microbiota of the A_V samples included *B. adolescentis* and *B. bifidum* (*P* < 0.05) (Fig. 5b and c). The percentage of *B. longum* was higher in the P_A group than in the control group, but there was no significant difference (Supplemental Table S4). The microbiota that were significantly lower in P_V and A_V samples compared to P_A samples were *B. adolescentis* and *B. bifidum* (*P* < 0.05) (Fig. 5d and e). These results showed that *B. longum* was more abundant in the control group. *B. adolescentis* and *B. bifidum* were more abundant in the P_A samples. *B. longum*, *B. adolescentis*, and *B. bifidum* may play a key role in the production of Uro-A since there was no significant difference in the Uro-A content of the fermentation broths of the control and P_A groups.

### Correlation analysis of EA metabolism and gut microbial structure during in vitro fermentation

To further investigate whether host-dependent changes in microbiota characteristics are associated with the production of certain urolithin metabolites, the Spearman correlation analysis was performed to explore the correlation between species and EA metabolites.

**Fig. 6:**
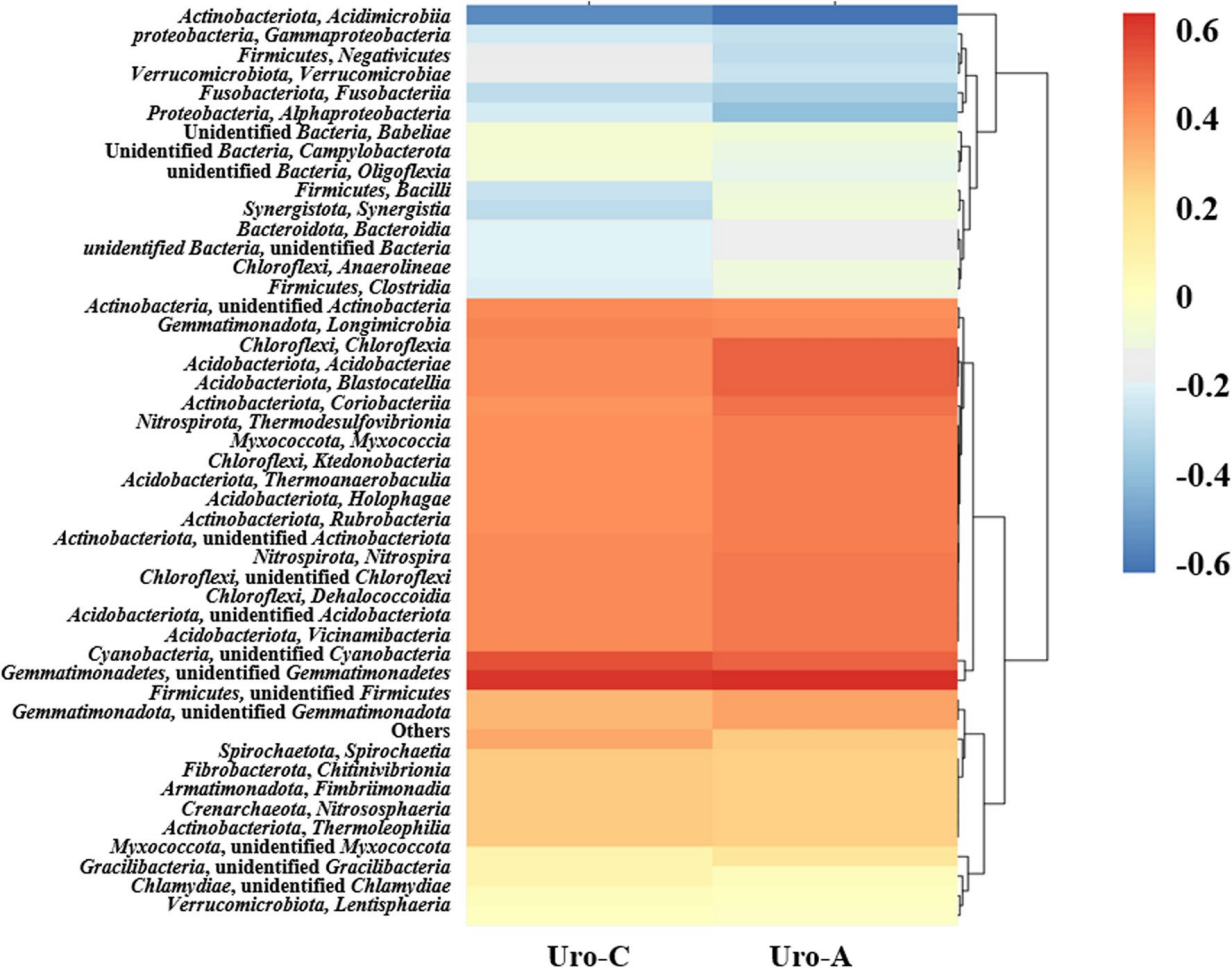
Heat map of the correlation between microbiome belonging to and urolithins formed by different phylum and class

At the phylum level, *Actinobacteria* and *Gemmatimonadetes* had the higher correlation with Uro-C and Uro-A production, and *Cyanobacteria* had the highest correlation with Uro-C (Fig. 6). At the class level, *Chloroflexi*, *Acidobacteria*, and *Blastocatellia* were closely associated with Uro-A production, and *Longimicrobia* was positively associated with Uro-C production.

## Discussion

Uro-A exhibits a multitude of health benefits, including antioxidant, anti-inflammatory, anticancer, antiobesity, and antiaging effects, and regulates estrogen receptors (D'Amico et al. 2021). Although urolithin-associated strains have been reported, research on specific gut microbiota that convert EA to Uro-A is limited. In this study, we found that an increase in the abundance of *B. longum*, *B. adolescentis*, and *B. bifidum* may enhance the utilization of EA and the production of Uro-A by the intestinal microbiome.

Only a few bacteria have been previously reported to produce Uro-A from EA. *B. pseudocatenulatum* INIA P815 was the first bacterium capable of producing Uro-A and Uro-B from EA (Gaya et al. 2018). In addition, the strains that have been reported to metabolize EA to produce Uro-A are *Streptococcus thermophilus* FUA329, *E. faecium* FUA027, and *L. garvieae* FUA009 (Beltran et al. 2018; Liu et al. 2022; Mi et al. 2022; Zhang et al. 2022c). These are all Gram-positive bacteria. However, no studies have shown that the various catalytic enzymes (acyl hydrolase, lactonase, decarboxylase, and catechol-dehydroxylase) that convert EA to produce Uro-A are specific for Gram-negative or Gram-positive bacteria. The composition of intestinal microbiome changes throughout a person’s life, and variations in the distribution of different microbial groups play a crucial role in maintaining the intestinal environment. Uro-A generation was significantly higher in the control and P_A group samples compared to the other groups. (Fig. 1b–d). In both sets of samples, the abundance of *B. longum*, *B. adolescentis*, and *B. bifidum* was dominated. The control group had a higher abundance of *Lachnospiraceae* and *Prevotellaceae* at the family level compared to the blank group. However, the abundances of *Lachnospiraceae* and *Prevotellaceae* were not significantly different between the control and the three experimental groups. Moreover, the abundance of *Actinobacteria* was higher in the P_A group than in the control group, and its abundance significantly increased compared to the other two experimental groups (Fig. 2b). Thus, the groups with higher Uro-A-producing levels might be composed of Gram-positive bacteria. Therefore, it is tentatively concluded that the Gram-positive bacterial microbiome in the fermentation broth plays a crucial role in metabolizing EA and producing urolithins.

Nowadays, genomic and metabolomic technologies are used for in vitro fermentation to study the interactions between metabolites and the gut microbiota (Yu et al. 2022). Studies conducted as early as the beginning of this century, which investigated in vitro EA metabolism by fecal microorganisms, have demonstrated that these microbes are capable of producing urolithins (Cerdaa et al. 2005). However, the concentrations of metabolites vary significantly among individuals, and there is no correlation found between the microbiota and EA metabolism. The 16S rRNA and transcriptome technology analyses have shown that the benefits of EA to the intestinal environment are partially influenced by host age, and that bacteria with significant changes in abundance are highly associated with genes related to phenolic metabolism (Duan et al. 2022). Besides, 16S rRNA sequencing could reveal the modulatory effects of polyphenol-rich green tea beverages on gut microbial composition and their metabolism. The results showed that green tea extract treatment altered the composition of the gut microbiota in patients with metabolic syndrome at the genus level and led to significant changes in the metabolic profile of the microbiota (Zhang et al. 2022b). At lower taxonomic levels, a high degree of interindividual variation is observed in the gut microbiota composition in terms of relative abundance and metabolic capacity, and these differences determine the types of microbial metabolites formed (Gan et al. 2023; Li et al. 2011). *B. longum*,* B. adolescentis*, and *B. bifidum* were more predominant in control and P_A samples. These bacteria are Gram-positive bacteria, and the EA metabolites Uro-A and Uro C were significantly higher compared to the other groups. We hypothesized that positive bacteria play a major role in the gut microbiome involved in EA transformation.

Urolithin is mainly produced in the distal colonic region, and garnet ETs enhance urolithin production and increase the levels of total bacteria such as *Faecalibacterium*, *Butyricicoccus*, *Odoribacter*, and *Butyricimonas* (Gonzalez-Sarrias et al. 2018). The microbiomes mentioned above differ from the relevant microbiomes that were studied in the present research. Furthermore, ETs could increase the abundance of *Bacteroides*, which is consistent with our results (Fig. 2a) (Garcia-Villalba et al. 2017).

*Bifidobacteria* levels are lower in infants exclusively fed formula milk than in breastfed infants, and longer breastfeeding also favors the proliferation of *Bifidobacteria* and *Weronococcus* while reducing the abundance of *Trichosporaceae*, *Rumexidae*, and other less prevalent microorganisms (Harmsen et al. 2000; Laursen et al. 2016). The bidirectional interaction between polyphenols and gut microbiota is well established. Growing evidence indicates that gut microbiota can determine the impact of polyphenols on an individual’s health (Gao et al. 2023; Tomás-Barberán et al. 2016). ET-produced urolithins in a rat intestinal inflammation model exhibited a positive modulation of *Lactobacillus*, *Bifidobacterium*, and *Enterobacteriaceae* (Larrosa et al. 2010). In our study, Spearman correlation analysis was used to analyze the relationship between urolithins and the abundance of microbiome in fecal samples of a 22-year-old volunteer. Uro-A can only be derived from Uro-C, while Uro-M5 and Uro-M6 are direct precursors of Uro-C (Iglesias-Aguirre et al. 2023). Besides, *Longimicrobia* has a high correlation with both urolithins. We conclude that *Longimicrobia* is hypothesized to play a role in the colon; *Chloroflexi*, *Acidobacteria*, and *Blastocatellia* further facilitate the action of catechol dehydroxylase. The enzymes and associated genes involved in the EA to Uro-A metabolic pathway are currently unknown. The combination of traditional culture techniques and genomic approaches may help to explore the molecular mechanisms of enzymes encoding specific metabolic activities.

Different microbial communities emerge as dominant species when their structure and function are stabilized, and they play unique and crucial roles. The species belonging to the same genus are closely linked to each other, but exchange of important information takes place between different genera to improve reactant utilization. Meanwhile, in the bidirectional interaction between EA and intestinal microbiome, both EA and urolithins can regulate the intestinal microbiome and thus mediate the action of (poly)phenols, in addition to ET catabolism by the intestinal microbiome to produce urolithins (Garcia-Villalba et al. 2022), but the underlying mechanisms need to be further explored.

In conclusion, we found that the Uro-A production capacity of gut microbiota treated with antibiotics polymyxin B sulfate and amphotericin B was consistent with that of gut microbiota without antibiotic treatment. Furthermore, the metagenomic analysis revealed that *B. longum*, *B. adolescentis*, and *B. bifidum* may play crucial roles in EA biotransformation. Our data provide insights into the interaction between EA phenolic compounds and gut microbiota, which could contribute to further development of gut bacteria as potential probiotics. In vivo experiments are also in our plan, and we will conduct in vivo experiments to further explore the key strains for transforming ellagic acid in the future.

## Supplementary Information

Below is the link to the electronic supplementary material.

Supplementary file1 (PDF 528 KB)

## Data Availability

The original contributions presented in the study are included in the article/supplementary material, and further inquiries can be directed to the corresponding authors.
