# Supplementary material for: In vitro conversion of ellagic acid to urolithin A by different gut microbiota of urolithin metabotype A
Source: Appl Microbiol Biotechnol. 2024 Feb 16;108:215. doi: 10.1007/s00253-024-13061-1 (PMC10873453; doi:10.1007/s00253-024-13061-1)
Supplement: Supplementary file 1 [file 253_2024_13061_MOESM1_ESM.pdf]

In vitro conversion of ellagic acid to urolithin A by different gut microbiota of urolithin metabotype A

Applied Microbiology and Biotechnology

Fuxiang He[1,2,3], Yingying Bian[3], Yaling Zhao[3], Mengjie Xia[1,2,3], Shu Liu[1,2,3], Jiajin Gui[1,2,3], Xiaoyue Hou[1,2,3,*], Yaowei Fang[1,2,3,*]

1. Jiangsu Key Laboratory of Marine Bioresources and Environment /Jiangsu Key Laboratory of Marine Biotechnology, Jiangsu Ocean University, Lianyungang 222005, China

2. Co-Innovation Center of Jiangsu Marine Bio-industry Technology, Jiangsu Ocean University, Lianyungang 222005, China

3. College of Ocean Food and Biological Engineering, Lianyungang 222005, China

* Correspondence authors:

Xiaoyue Hou: 2020000057@jou.edu.cn.

Yaowei Fang: 2007000027@jou.edu.cn.

Supplementary Materials:

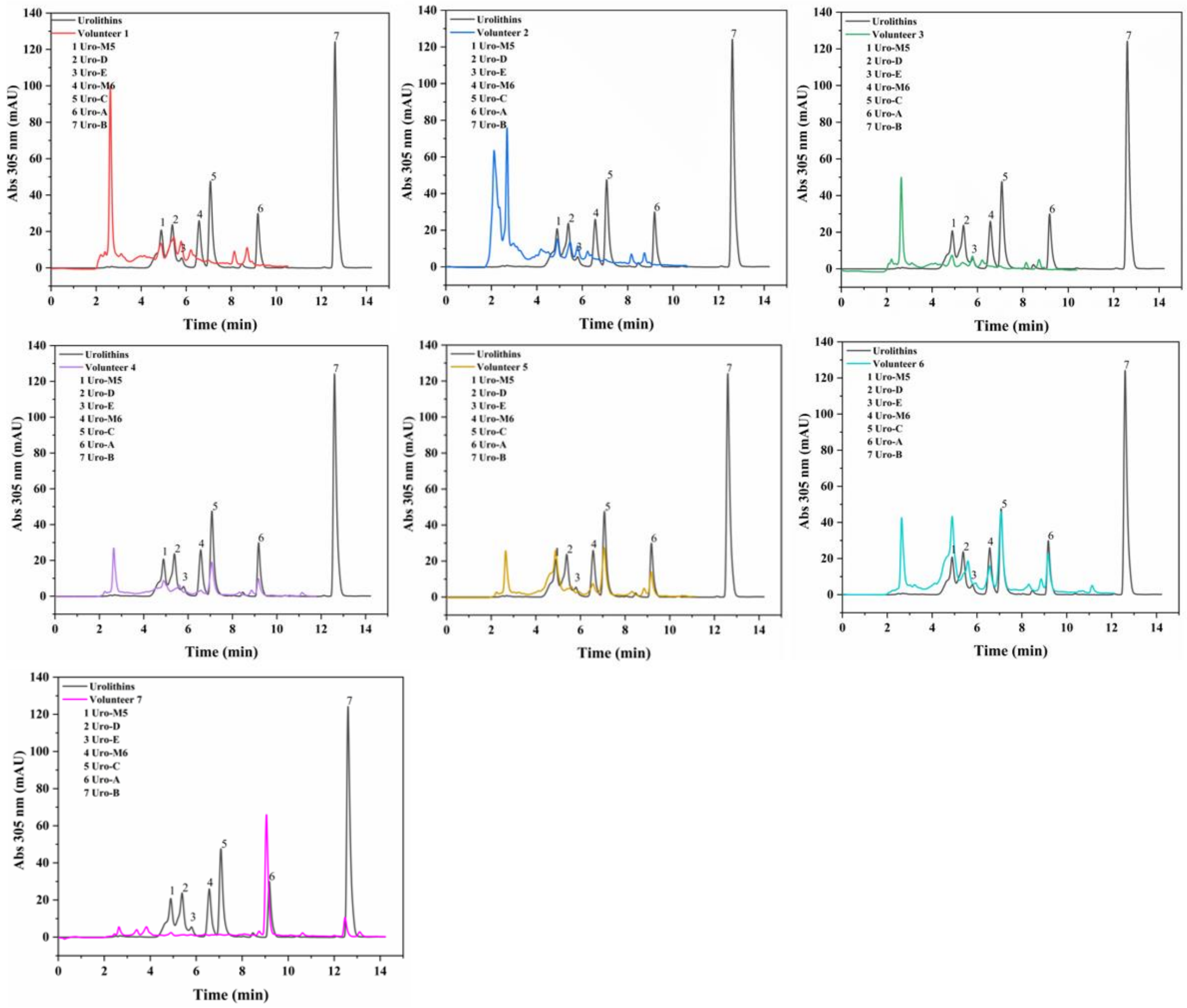

Fig. S1. HPLC identification of urolithin metabolic types in 7 volunteers. Volunteers 1-3 are UM-0, 4-6 are UM-A and 7 is UM-B.

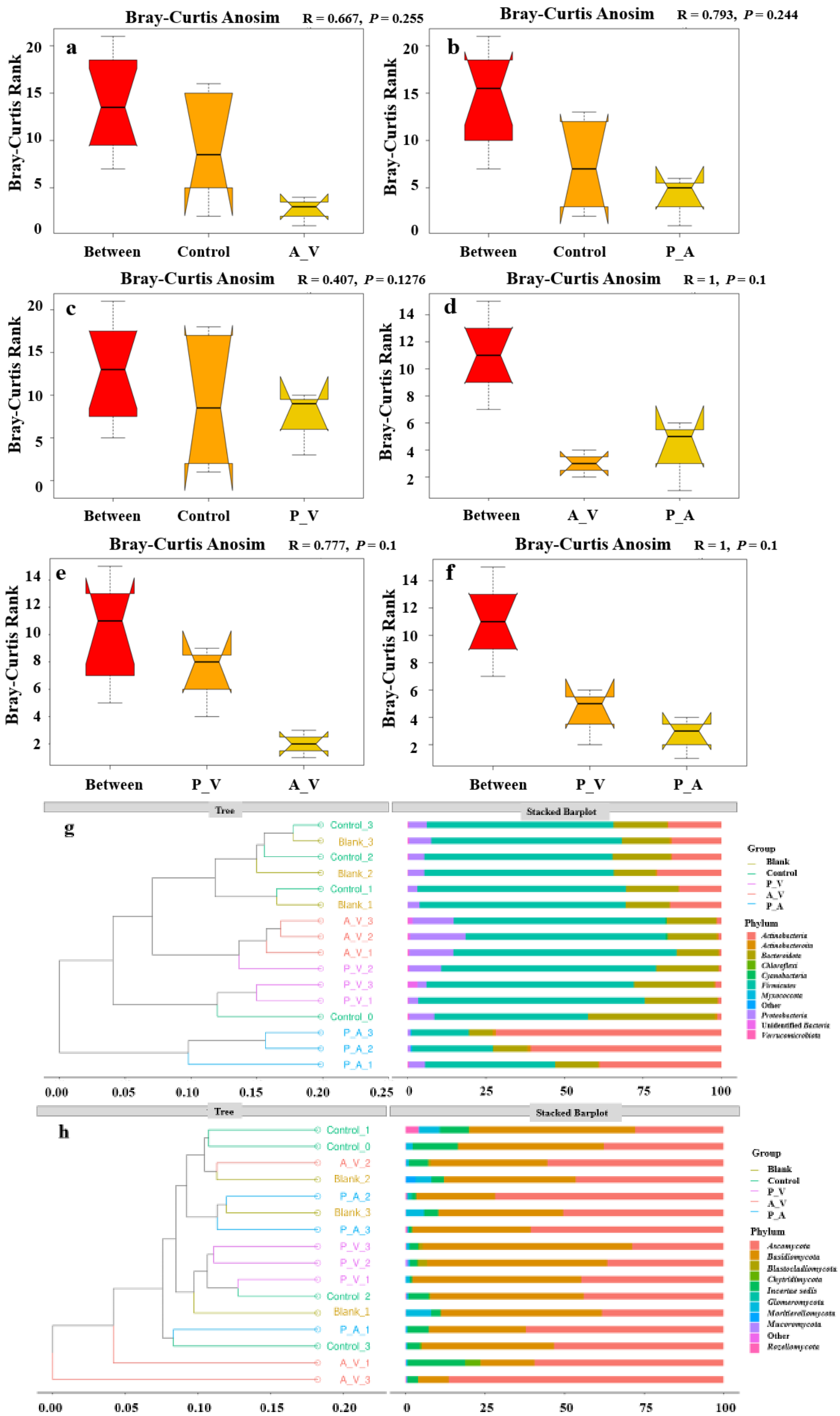

Fig. S2. Inter- and intragroup differences, the similarity analysis (Anosim). (a–f) Similarity analysis of different intestinal microbiota. (g–h) Hierarchical clustering analysis of different intestinal microbiota.

Table S1. LC-MS/MS (G2-XS QTOF) characteristics of 15 μM urolithins and EA detected in the present work or used as standards.

**Standards**	**Formula**	**[M-H] (m/z)**	**Retention time (min)**	**Area**
Urolithin A	$C_{13}H_8O_4$	227.0346	11.47	556.1195
Urolithin C	$C_{13}H_8O_5$	259.0245	8.15	1060.1349
Urolithin M5	$C_{13}H_8O_7$	275.0192	6.10	7889.9680
Urolithin M6	$C_{13}H_8O_6$	300.9982	7.61	2980.3081
Iso Urolithin A	$C_{13}H_8O_4$	243.0296	8.89	667.8921

Table S2. Intestinal microbiome richness in all the samples. Metagenomic bacterial taxonomic distribution based on relative abundance of clean metagenomic reads at family level.

**Taxonomy**	**Blank**	**Control**	**P_V**	**A_V**	**P_A**
Lactobacillaceae	0.001993	0.001715	0.063682	0.008576	0.006859
Rhodobacteraceae	0.029337	0.027298	0.016808	0.026129	0.008082
Enterobacteriaceae	0.008626	0.006959	0.029864	0.108445	0.002219
Prevotellaceae	0.025962	0.111025	0.128654	0.037561	0.037025
Bacteroidaceae	0.095933	0.094424	0.072015	0.073288	0.041681
Ruminococcaceae	0.100657	0.098129	0.10971	0.033633	0.099124
Veillonellaceae	0.189873	0.130433	0.037703	0.112524	0.015954
Selenomonadaceae	0.143611	0.119975	0.117825	0.399285	0.006323
Lachnospiraceae	0.14377	0.190198	0.301266	0.089233	0.113554
Bifidobacteriaceae	0.170527	0.113399	0.009505	0.007487	0.567065
Others	0.089711	0.106446	0.112968	0.103839	0.102114

Table S3. Intestinal microbiome heat map of species abundance in all the samples. Metagenomic bacterial taxonomic distribution based on relative abundance of clean metagenomic reads at genus level. The values corresponding to the heat map are relative quantitative data standardized by Z-Score.

**Short taxnomy**	**P_V**	**Control**	**Blank**	**A_V**	**P_A**
Enterococcus	-0.77443	-0.4869	0.683944	-0.83575	1.41313
Bifidobacterium	-0.71114	-0.26089	-0.0133	-0.71989	1.705225
Ruminococcus	0.950331	-0.38662	-0.80992	-0.94017	1.186381
Alistipes	0.475636	-0.50098	-0.76026	-0.75529	1.540889
Barnesiella	1.335383	-0.14368	-0.97477	-0.89026	0.673326
Unidentified *Clostridia*	1.391566	-0.32126	-0.84446	-0.8945	0.668652
Fusicatenibacter	1.700032	0.022878	-0.42627	-0.85691	-0.43973
Lachnospira	1.685967	0.016041	-0.56984	-0.87563	-0.25654
Lachnoclostridium	1.560053	0.338004	-0.59165	-1.00576	-0.30065
Unidentified *Prevotellaceae*	1.255609	0.908047	-0.88128	-0.6419	-0.64048
Agathobacter	1.602977	0.247992	-0.22819	-0.80168	-0.8211
Others	1.446624	0.528233	-0.3031	-0.57225	-1.0995
Paraprevotella	1.618875	0.267248	-0.60526	-0.39428	-0.88658
Unidentified *Lachnospiraceae*	1.531569	0.490239	-0.80307	-0.49452	-0.72422
Desulfovibrio	1.626541	-0.59526	-0.66828	0.312235	-0.67523
Dorea	1.7245	-0.50676	-0.40345	-0.77794	-0.03634
Weissella	1.774317	-0.50348	-0.51954	-0.22373	-0.52757
Roseburia	1.763337	-0.19978	-0.39025	-0.51554	-0.65776
Subdoligranulum	1.200427	0.580674	0.178031	-1.34767	-0.61146
Blautia	1.015028	0.251897	0.365323	-1.66485	0.032606
Bacteroides	-0.15696	0.861534	0.930144	-0.0991	-1.53562
Allisonella	-0.86527	0.465799	1.366791	0.107259	-1.07458
Ruegeria	-0.5224	0.623337	1.005248	0.373244	-1.47943
Ilumatobacter	-0.93217	0.486349	0.902305	0.765786	-1.22227
Unidentified *Erysipelotrichales*	-0.69657	0.620094	1.469611	-0.70378	-0.68935
Faecalibacterium	-0.21881	0.666112	1.285792	-1.28687	-0.44622
Unidentified *Oscillospiraceae*	1.031377	1.047442	-0.48945	-0.38449	-1.20488
Coprococcus	0.576551	1.423261	-0.29111	-0.63374	-1.07496
Coprobacter	-0.28371	1.478064	-0.14069	0.234724	-1.28839
Raoultella	-0.02414	-0.55375	-0.48659	1.734207	-0.66973
Citrobacter	-0.05643	-0.53128	-0.56483	1.744891	-0.59235
Unidentified *Enterobacteriaceae*	-0.15591	-0.50486	-0.47605	1.761725	-0.62491
Megamonas	-0.27229	-0.25763	-0.09487	1.664302	-1.03951
Parabacteroides	-0.34989	-0.32013	-0.35928	1.762905	-0.7336
Dialister	-0.08075	0.236116	-0.14667	1.394972	-1.40366

Table S4. Intestinal microbiome richness in all the samples. Metagenomic bacterial taxonomic distribution based on relative abundance of clean metagenomic reads at species level.

**Taxonomy**	**Blank**	**Control**	**P_V**	**A_V**	**P_A**
Metagenome	0.013148	0.013743	0.019756	0.004112	0.012788
Weissella cibaria	0.000636	0.001024	0.055433	0.007671	0.000435
Bacteroides plebeius	0.021883	0.02177	0.009397	0.006549	0.007722
Bacteroides vulgatus	0.014019	0.012813	0.022654	0.033072	0.010293
Bifidobacterium. bifidum	0.009648	0.006193	0.004171	0.001172	0.073874
Ruminococcus. sp. N15 MGS 57	0.011557	0.017059	0.044746	0.011574	0.054269
Allisonella histaminiformans	0.064444	0.039847	0.006407	0.032142	0.000904
Bifidobacterium longum	0.112331	0.074877	0.002454	0.003283	0.131945
unidentified Prevotellaceae	0.023985	0.108035	0.124374	0.035233	0.035249
Bifidobacterium adolescentis	0.043105	0.029634	0.00242	0.002311	0.30434
Others	0.685242	0.675004	0.708189	0.86288	0.368181
